# Mr. Keyser's Proposal

**Published:** 1921-06-04

**Authors:** 


### MR. KEYSER'S PROPOSAL.

Much interest will be aroused by Mr. Leonard Keyser's proposals in *The Times* of Tuesday last. When, as Treasurer of the Royal Hampshire County Hospital, he first proposed his scheme for what is now known as insurance for free hospital treatment to regular subscribers of 2*d*. a week for adults or 6*d*. a week for a whole family, we described it in detail in these columns. It was recommended to the meetings, which he addressed in the statement, "Keep the hospital when you are well and we will treat you when you are ill," and has had encouraging results, not only in Hampshire.

Mr. Keyser asks why a similar plan cannot be adopted in London? For more than a year we have asked the same question. Some attempt has been made here and there, but nothing like general results have been gained. Have they even been attempted? If not, we should like, in no captious spirit, to inquire why? Mr. Keyser suggests that the organisation required would be very great; but one cannot think that it would be overwhelming. The point, at all events, is that some such scheme as this is the only one in the field which, if adopted, could solve our difficulties on voluntary principles. What we now want to hear is a statement of the *practical difficulties* in the way. Perhaps the Chairmen of the London hospitals, to whom we have so often recommended virtually this very plan, will enlighten us. And that there may be no mistake about it we will end by quoting Mr. Keyser's own words:

> The requirements seem to me to be, first and principally, the co-operation of all the hospitals in the London area; secondly, committees in each district; thirdly, the assistance and goodwill of all employers of labour. *Think of 2d. a week from every wage-earner in London!*

Apart from the italics, which we have added, Mr. Keyser's scheme differs from ours chiefly in that his offers a *quid pro quo*. We hereby invite Lord Hambleden, Lord Knutsford, and other chairmen to propound their views of its practical difficulties. For in this way the scheme can be judged in a fair light, and perhaps a residuum may be found for a joint endeavour. Mr. Coles and Mr. Inman, of the Saturday Fund, discussed it a year ago.

